# RenSeq-derived SNPs used in mapping resistance to *Phytophthora sojae* and three *Pythium* spp. in multiple soybean RIL populations

**DOI:** 10.1007/s11032-026-01686-1

**Published:** 2026-06-13

**Authors:** Elizabeth M. Clevinger, Ruslan Biyashev, Brian Hodge, Alison E. Robertson, Leah K. McHale, Anne E. Dorrance, M. A. Saghai Maroof

**Affiliations:** 1 School of Plant and Environmental Sciences, Virginia Tech, Blacksburg, VA USA; 2 Department of Plant Pathology, Center for Soybean Research, The Ohio State University, Wooster, OH USA; 3 Department of Plant Pathology, Entomology and Microbiology, Iowa State University, Ames, IA USA; 4 Department of Horticulture and Crop Science, The Ohio State University, Columbus, OH USA

**Keywords:** QDRL, Soybean, *Pythium*, *Phytophthora sojae*, RenSeq

## Abstract

*Phytophthora sojae* and *Pythium* spp. cause important root diseases of soybeans resulting in millions of dollars of loss for producers every year. Our previous studies on mapping *Rps* (Resistance to *Ph. sojae*) genes and quantitative disease resistance loci (QDRL) towards *Ph. sojae* and *Pythium* species, respectively, identified key regions for resistance to these diseases on chromosomes 3, 6, 7, 8, 13, 14 and 18. The development of *R* gene enrichment and sequencing (RenSeq) have allowed for the expedited discovery of new nucleotide-binding, leucine-rich repeat (NLR)-encoding genes. This study focused on exploring the co-localization of *Rps* loci or QDRL regions with single nucleotide polymorphism (SNP) markers derived from sequences identified from a RenSeq study of germplasm varying for resistance to *Ph. sojae* or *Pythium* species. Another aspect of this study was to saturate the QDRL regions on chromosomes 6, 8 and 14 for resistance to multiple *Pythium* species utilizing conventional molecular and SNP markers developed from RenSeq analysis. Nineteen RenSeq SNP markers were selected for evaluation in six RIL populations related to resistance to *Ph. sojae* and/or *Pythium* as allele-specific KASP markers. Most RenSeq SNP markers co-localized within the *Rps* loci or QDRL regions in these RIL populations. The development of RenSeq SNPs and their co-localization with *Rps* loci or QDRL regions indicate their potential use in designing resistance gene-specific markers and their application for marker-assisted selection in soybean resistance breeding programs.

**Supplementary Information:**

The online version contains supplementary material available at 10.1007/s11032-026-01686-1.

## Introduction

Soybean (*Glycine max* (L.) Merr.) is a major legume crop grown worldwide. In the United States in 2024, soybean was planted on more than 87 million production acres with a value of $51 billion (http://www.soystats.com). Yield can be suppressed by multiple diseases. The average loss in 2024 in the United States to soybean seedling diseases, such as those caused by *Pythium* spp., was over $235 million, while losses due to Phytophthora root and stem rot (causal agent *Phytophthora sojae*), (Kaufmann and Gerdemann) were over $203 million (Crop Protection Network 2025). Recent surveys of both *Pythium* and *Ph. sojae* have shown a diversity of species and pathotypes, respectively in the United States (Rojas et al. 2017; Dorrance 2018; Matthiesen et al. 2021; Navarro et al. 2021; Hebb et al. 2022; McCoy et al. 2023). While a single species of *Pythium* can cause disease, several species are often isolated from an individual plant (Rizvi and Yang 1996; Dorrance et al. 2004; Broders et al. 2007; Zitnick-Anderson and Nelson 2015). In our previous studies, mapping QDRL towards multiple species of *Pythium* and *R*-gene mediated resistance towards isolates of *Ph. sojae* (*Rps* genes) in six soybean recombinant inbred line (RIL) populations, we identified several key chromosomal areas that encompassed large regions of the genome (Table 1).

**Table 1:** Summary of previously mapped chromosomal locations *Rps* genes or quantitative disease resistance loci (QDRL) for resistance towards *Phytophthora sojae* and *Pythium* species, respectively, in multiple recombinant inbred line populations

Population	Chromosome	Rps gene or QDRL	Generation Assayed	Population Size	Disease caused by	Citation
PI 399079 ×Williams	3, 7, 13, 18	*Rps* gene	F_7,_ F_9_	211	*Phytophthora sojae*	Clevinger et al. 2025
PI 408132 ×Williams	3, 7, 13	*Rps* gene	F_7,_ F_9_	142	*Phytophthora sojae*	
PI 407985 ×Williams	3, 13, 18	*Rps* gene	F_7_, F_8_, F_9_	192	*Phytophthora sojae*	Clevinger et al. 2024
PI 408029 ×Williams	3, 13	*Rps* gene	F_9_	188	*Phytophthora sojae*	
PI 408097 ×Williams	3, 13, 18	*Rps* gene	F_8_, F_9_, F_10_, F_11_	312	*Phytophthora sojae*	
PI 424477 ×Williams	3, 13	*Rps* gene	F_7_, F_9_	163	*Phytophthora sojae*	
PI 408029 ×Williams	6	QDRL	F_8_	188	*Pythium sylvaticum*,* Py. irregulare*	Clevinger et al. 2021
PI 408029 ×Williams	14	QDRL	F_8_	188	*Pythium sylvaticum*	
PI 408097 ×Williams	8	QDRL	F_10_	312	*Pythium sylvaticum*,* Py. irregulare*,* Py. torulosum*	

The development of resistance gene (*R* gene) enrichment and sequencing (RenSeq) has allowed the expedited discovery of novel NLR (nucleotide-binding leucine-rich repeat domains) encoding genes or alleles present in unique germplasm (Jupe et al. 2013). NLR-encoding genes generally function in race-specific disease resistance (McHale et al. 2006) but have also been shown to be associated with QDR (Gou et al. 2023). Regions in the soybean genome that harbor NLR genes are very complex with multiple copies covering large spans of the genome (McHale et al. 2006, 2012; Kang et al. 2012). Recently, Hodge et al. (2025) utilized RenSeq to identify and compare sequence variants of NLR-encoding genes from 20 soybean accessions with resistance to *Ph. sojae*. Focusing on 31 NLR gene models from the “Williams 82” genome assembly (v4.0) which were located nearby *Rps* loci, NLR variants from 28 soybean accessions displaying diverse sources of resistance to *Ph. sojae* were reported.

The present study used single nucleotide polymorphism (SNP) markers from sequences enriched for NLR-encoding genes, based on RenSeq output from accessions with resistance to *Ph. sojae* on four soybean chromosomes (Hodge et al. 2025) and other sequence derived DNA markers, to (i) explore the correspondence or co-localization of RenSeq-derived markers to previously mapped *Rps* (Resistance to *Ph. sojae*) loci regions on chromosomes 3, 7, 13 and 18 (Clevinger et al. 2024, 2025) and (ii) saturate QDRL regions with additional conventional DNA markers and RenSeq-derived SNPs on chromosomes 6, 8 and 14 where multiple *Pythium* spp. map. This study expands on previous studies by Clevinger et al. (2021; 2024; 2025) and Hodge et al. (2025) towards the development of gene-specific DNA markers for marker-assisted selection focused on soilborne pathogens in soybean breeding programs.

## Materials and methods

Genetic materials for this study included six advanced generation RIL populations, constructed and developed in Blacksburg, Virginia, that were previously used to map novel *Rps* genes and *Pythium* QDRL (Table 1). Also from previous studies, the phenotypic data was used in these traits including: seed rot severity (SRS) and percentage of symptomatic seed (ROTS) following inoculation with Pythium spp. (Clevinger et al. 2021) and single and d combined isolate inoculum from cross of Williams with PI 399079, PI 407985, PI 408097, PI 408132, and PI 424477 (Table 1) (Clevinger et al. 2024, 2025).

The five *Ph. sojae* mapping populations were genotyped previously (Clevinger et al. 2024, 2025). In this study, RenSeq-derived SNPs were developed, RIL genotyped as described below, and used for mapping. Hodge et al. (2025) identified several candidate NLR encoding resistance genes in the previously mapped regions which confer resistance to *Phytophthora sojae*. For this study, we specifically selected ten NLR genes (Table 2, column 1) from chromosomes 3, 7, 13 and 18 and three from within the gene, *Glyma.07G077700* (Table 2 in Hodge et al. 2025). Both RenSeq-derived SNPs and additional molecular markers were employed to better saturate the QDRL regions of interest for resistance to *Pythium* spp. on chromosomes 6, 8 and 14 (Clevinger et al. 2021) by (1) polymorphic SSR markers in the QDRL regions (2) SNP markers selected from the 50 K SNP database and (3) using the same *Ph. sojae* RenSeq variant call files, potential polymorphic SNPs were identified on chromosomes 6, 8 and 14 (Supplementary Table 1, column 1). Of the seven SNPs identified this way (Supplementary Table 1), two on chromosome 14, resided within predicted disease resistance-related loci, the other SNPs were not canonical NLRs, but were identified by the RenSeq enrichment assay. To increase the number of markers in the QDRL regions on chromosomes 6, 8 and 14, SNPs were selected from the soybean 50 K SNP database (https://www.soybase.org/tools/snp50k/). These were chosen based on their polymorphism between the parental lines of each RIL population and their location on the chromosome (Supplementary Table 1, columns C, D, E).

**Table 2:** Summary of the RenSeq-derived KASP markers and their corresponding gene models for chromosomes 3, 7, 13 and 18 that are associated with *Rps* gene mediated resistance to *Phytophthora sojae* in RIL populations derived from crosses of PIs (Plant Introductions) and Williams (Wm)

Gene Model*	RenSeq SNP ID	PI399079 × Wm	PI407985 × Wm	PI408097 × Wm	PI408132 × Wm	PI424477 × Wm
*Glyma.03G043000*	3G043000_8029	mapped	-	-	-	-
*Glyma.03G043000*	3G043000_1509	-	mapped	-	-	mapped
*Glyma.03G038800*	3G038800_9619	-	mapped	-	mapped	mapped
*Glyma.07G077700*	7G077700_9147	mapped	-	-	-	-
*Glyma.07G077700*	7G077700_1360	mapped	-	-	-	-
*Glyma.13G187900*	13G187900_9025	-	mapped	mapped	mapped	-
*Glyma.13G188300*	13G188300_4376	-	mapped	mapped	mapped	-
*Glyma.18G280300*	18G280300_0296	-	mapped	mapped	-	-
*Glyma.18G280366*	18G280366_5883	-	mapped	mapped	-	-
*Glyma.18G281600*	18G281600_7595	mapped	mapped	mapped	-	-
*Glyma.18G281700*	18G281700_6714	mapped	mapped	mapped	-	-
SF273_U8800	U8800_608	mapped	mapped	mapped	-	-

*Williams82 genome assembly v4.0

-indicates that a SNP was not mapped in that population due to being monomorphic and/or not having a gene of interest on that chromosome

The DNA template sequences that were at least 100 bp long (50 + mer sequences each adjacent to the SNP on both sides) from candidate resistance genes were provided to 3CR Bioscience (Essex, UK) for primer design and synthesis. The resultant KASP primers were verified on DNA samples from the two parental lines of each RIL population and those showing clear unambiguous separation between calling genotypes were used for mapping the RenSeq variants All KASP primers are listed in Supplementary Table 1.

For KASP genotyping, PACE 2.0 Genotyping Master Mix (3CR Bioscience, Essex UK) was used following the manufacturer’s protocol with DNA template content of 30–45 ng per each genotyping reaction. Once the PCR reaction was complete, fluorescent signal data were collected using a FLUOstar Omega (BMG Labtech Inc., Cary, NC, USA) plate reader. The fluorescent signal data were analyzed using KlusterCaller software (version 3.4.1.39) by LGC Genomics (Herts, UK). In total, 12 RenSeq SNPs (Table 2) were selected for mapping in five RIL populations as allele-specific KASP markers (Supplementary Table 1, column 1).

The KASP marker data developed in this study were incorporated into the existing phenotypic and genotypic data of each of the RIL populations using JoinMap 4.0 (van Ooijen 2006) based on an LOD threshold of 3.0 and a maximum recombination frequency of 0.5 for the original grouping. Linear order of markers and their relative distances from each other on individual chromosomes were calculated by using the maximum likelihood algorithm and Kosambi mapping function (Kosambi 1944). The same phenotypic data from our previously reported studies (Clevinger et al. 2021, 2024, 2025) were used for the mapping of *Rps* genes towards *Ph. sojae* isolates and QDRL mapping for multiple *Pythium* species utilizing MapQTL 5 software (van Ooijen 2004) and was carried out as described in Clevinger et al. (2025).

### *Rps* genes, NLR for *Phytophthora sojae*

#### Chromosome 3

**Fig. 1:**
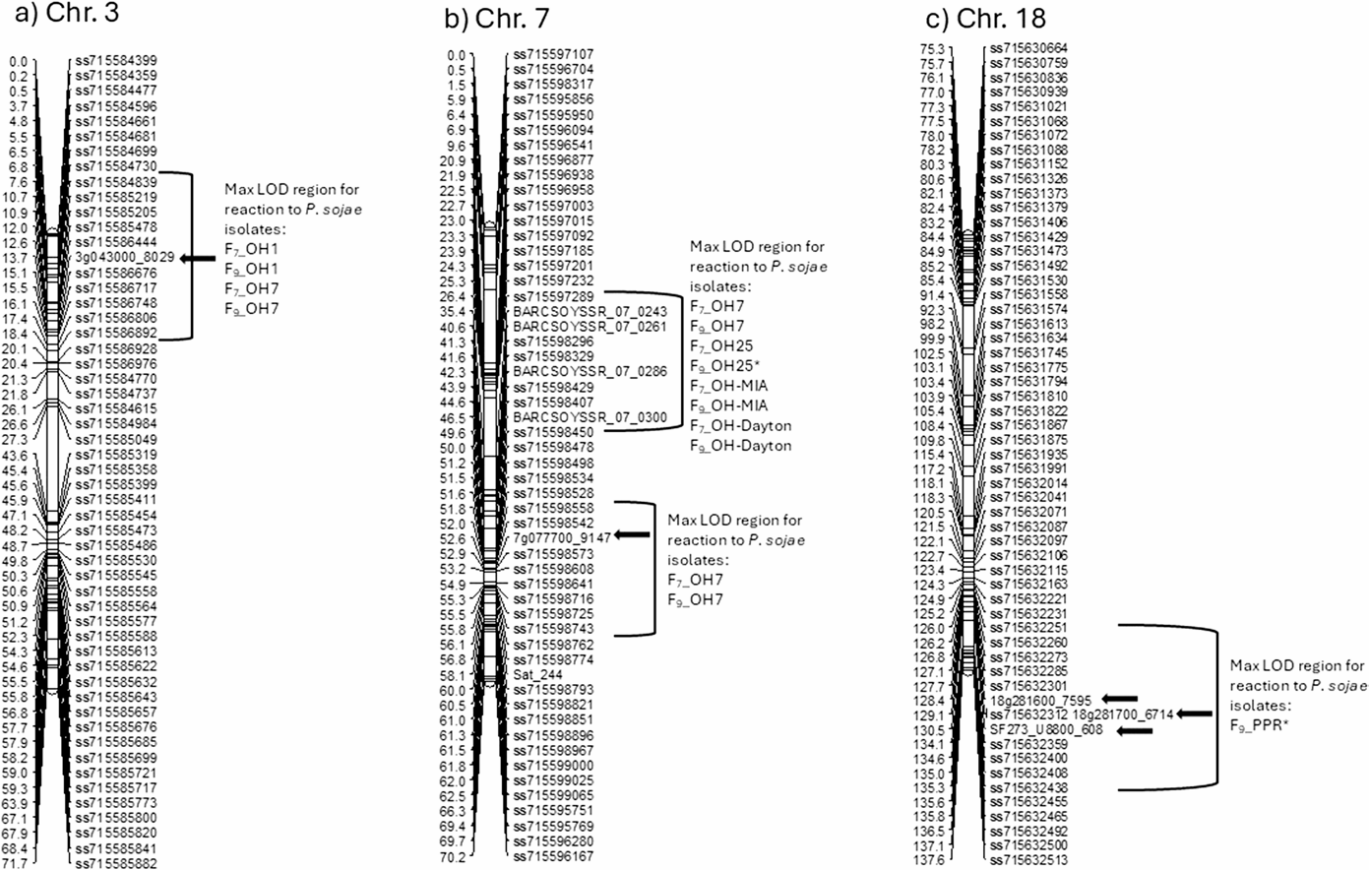
*Rps* gene mediated resistance mapping toward *Ph. sojae* in PI 399079 × Williams. Markers within the maximum logarithm of odds (LOD) region for reaction to specific isolates are denoted by brackets on the right side of each chromosomal map. A black arrow denotes the RenSeq-derived SNP mapped to that maximum LOD region. **a** Chromosome 3 regional map for reaction to isolates OH1 and OH7. **b** Chromosome 7 regional map for the response to isolates: OH7, OH25, OH-MIA, OH-Dayton. **c** Chromosome 18 regional map for reaction to combined isolates of *Ph. sojae* (PPR). Isolates designated with an “*” indicate that disease screening results from two replications were combined into a single dataset

Two genes, *Glyma.03G038800* and *Glyma.03G043000*, were proposed previously by Hodge et al. (2025) as candidate genes for *Rps1c*. Since many *R* genes have been mapped to this region on chromosome 3, it has been proposed that there may be several additional *R* alleles or paralogs located at this locus on chromosome 3 (Dorrance 2018; Lin et al. 2022). It should be noted that *Rps1c* per se is derived from Arksoy and not proposed to be the gene in these PIs (Demirbas et al. 2001; Mueller et al. 1978). Three RenSeq-derived SNPs including 3G043000_8029, 3G043000_1509 and 3G038800_9619 designed from these two candidate genes, were mapped in one or more of the five RIL populations (Table 2) based on parental polymorphism. The RenSeq marker, 3G043000_8029, was polymorphic only between PI 399079 and Williams and therefore mapped only in the PI 399079 × Williams population (Table 2; Fig. 1a).

**Fig. 2:**
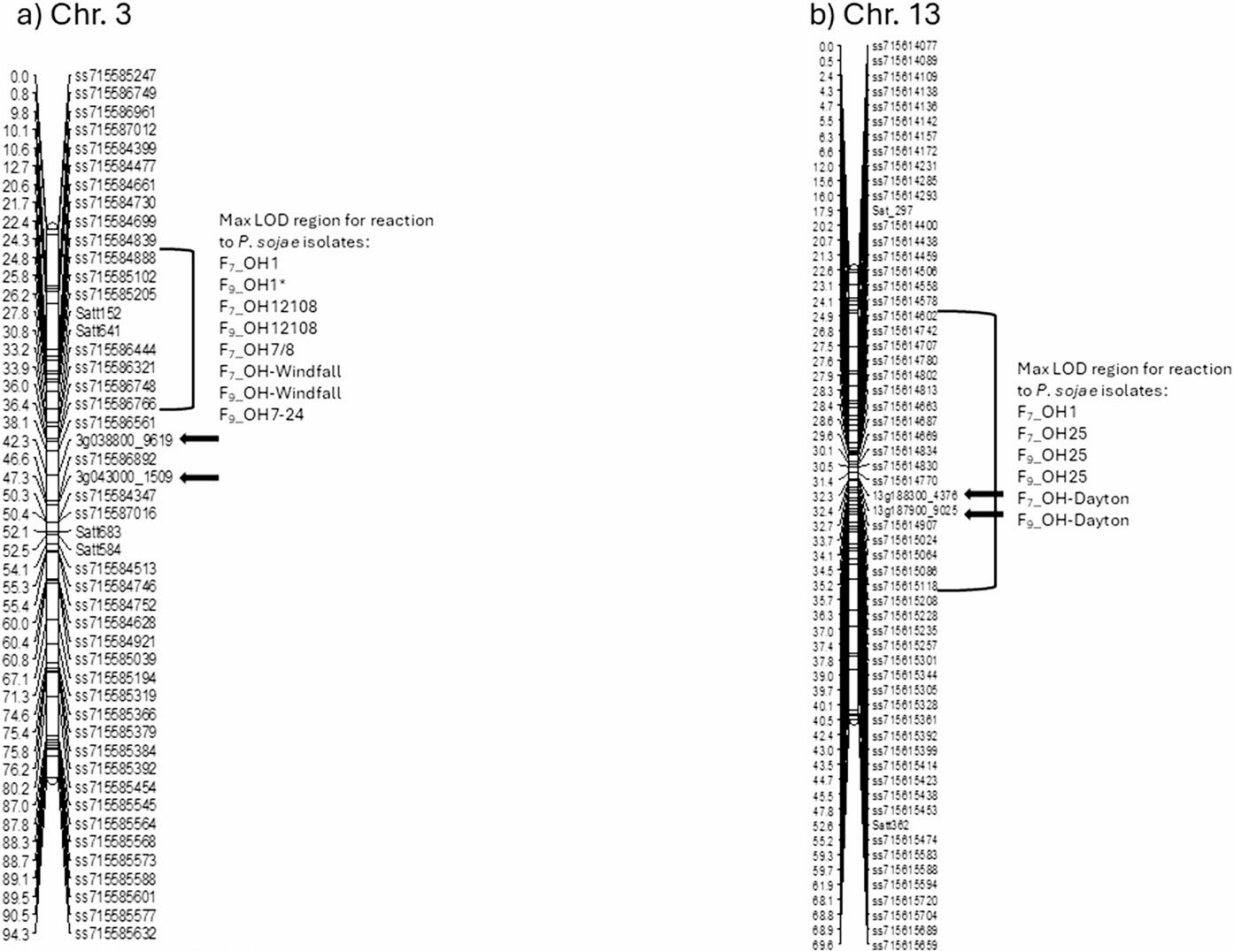
*Rps* gene mediated resistance mapping toward *Ph. sojae* in PI 408132 × Williams. Markers within the maximum logarithm of odds (LOD) region for reaction to specific isolates are denoted by brackets on the right side of each chromosomal map. A black arrow denotes the RenSeq-derived SNP mapped to that maximum LOD region or right outside the region. **a** Chromosome 3 regional map for reaction to isolates: OH1, OH12108, OH7/8, OH7-24, and OH-Windfall. **b** Chromosome 13 regional map for reaction to isolates: OH1, OH25, OH-Dayton. Isolates designated with an “*” indicate that disease screening results from two replications were combined into a single dataset

**Fig. 3:**
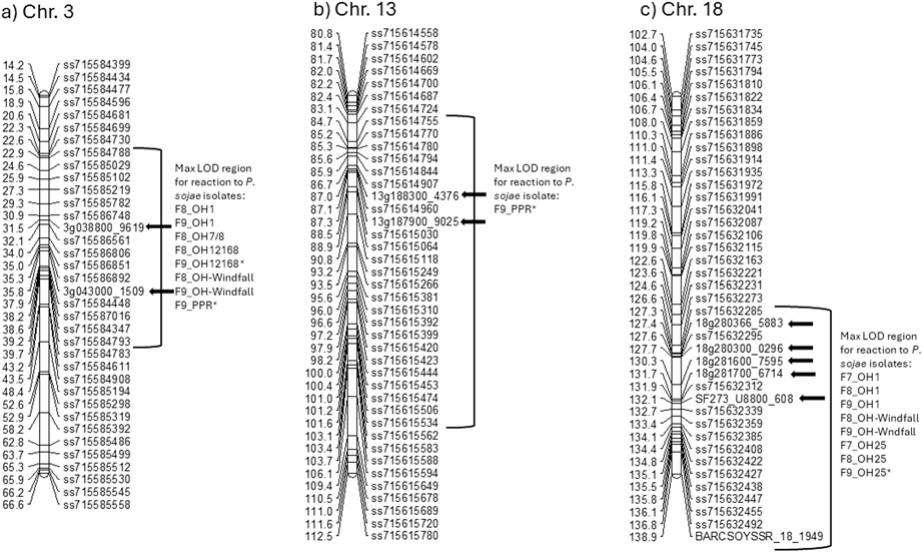
*Rps* gene mediated resistance loci mapping toward *Ph. sojae* in PI 407985 × Williams. Markers within the maximum logarithm of odds (LOD) region for reaction to specific isolates are denoted by brackets on the right side of each chromosomal map. A black arrow denotes the RenSeq-derived SNP mapped to that maximum LOD region. **a** Chromosome 3 regional map for reaction to isolates: OH1, OH7/8, OH12168, OH-Windfall and combined isolates of *Ph. sojae* (PPR). **b** Chromosome 13 regional map for reaction to isolate: PPR. **c** Chromosome 18 regional map for reaction to isolates: OH1, OH25 and OH-Windfall. Isolates designated with an “*” indicate that disease screening results from two replications were combined into a single dataset

**Fig. 4:**
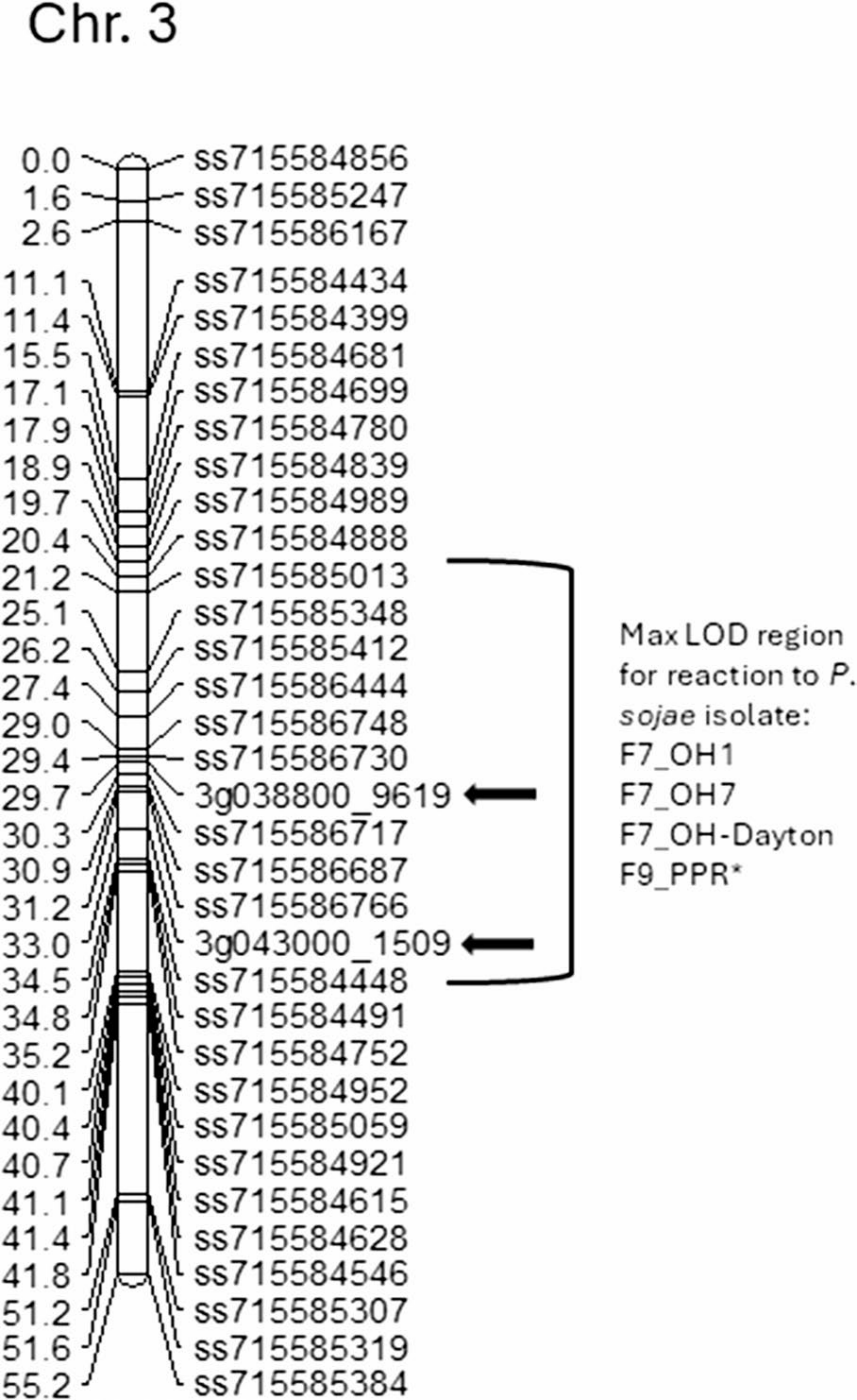
*Rps* gene mediated resistance loci mapping toward *Ph. sojae* in PI 424477 × Williams on chromosome 3 for reaction to isolates: OH1, OH7, OH-Dayton and combined isolates of *Ph. sojae* (PPR). The markers within the maximum logarithm of odds (LOD) region for reaction to specific isolates are denoted by brackets on the right side of the chromosomal map. A black arrow denotes the RenSeq-derived SNP mapped to that maximum LOD region. Isolates designated with an “*” indicate that disease screening results from two replications were combined into a single dataset

Three of the KASP RenSeq-derived markers mapped to the *Rps* loci in three populations. The marker, ss715586444, at the maximum LOD position for resistance towards *Ph. sojae* in PI 399079 was 1,054,587 bp away from the RenSeq marker, 3G043000_8029. Two RenSeq markers, 3G038800_9619 and 3G043000_1509, detected polymorphism between multiple parental lines and were mapped in PI 408132 × Williams, PI 407985 × Williams, and PI 424477 × Williams (Table 2; Figs. 2a, 3a and 4). The two RenSeq-derived SNPs for chromosome 3 in the PI 408132 population mapped 373,677 bp below the maximum LOD region for *Ph. sojae* (Fig. 2a).

#### Chromosome 7

Previous mapping (Clevinger et al. 2025) and cluster analysis (Hodge et al. 2025) proposed a novel *Rps* locus on chromosome 7 in the PI 399079 × Williams population. Unique variants from the NLR allele of *Glyma.07G077700* from PI 399079 were distinctly different than those from susceptible genotypes making it a good candidate for further investigation. One of these variants, the SNP located at position 9147, was specific for PI 399079 (Hodge et al. 2025). An additional SNP was observed at position 1360 in *Glyma.07G077700* (Supplementary Table S1). These two SNPs co-segregated to the same location on chromosome 7 in this population of 211 RILs. Only the marker at position 9147 is shown in Fig. 1b. The identified genetic map position agreed with the physical location of *Glyma.07G077700* (Wm82.a4) as well as with the maximum LOD for reaction to *Ph. sojae* on chromosome 7 (Clevinger et al. 2025) (Fig. 1b). This *Rps* gene appears to be below the region previously reported with *Rps11* (Wang et al. 2021).

#### Chromosome 13

**Fig. 5:**
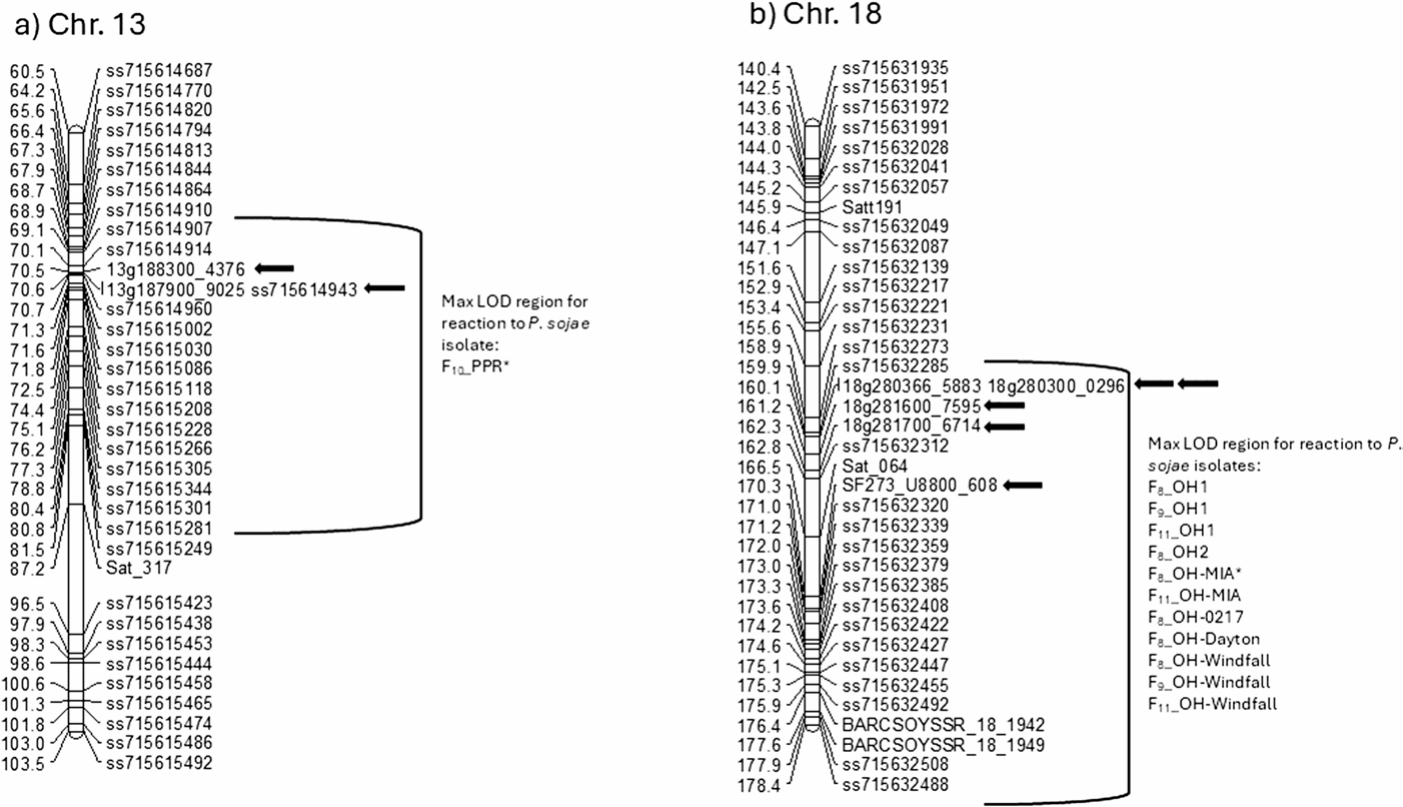
*Rps* gene mediated resistance loci mapping toward *Ph. sojae* in PI 408097 × Williams. The markers within the maximum logarithm of odds (LOD) region for reaction to specific isolates are denoted by brackets on the right side of each chromosomal map. A black arrow denotes the RenSeq-derived SNP mapped to that maximum LOD region. **a** Chromosome 13 regional map for the reaction to the combined isolates of *Ph. sojae* (PPR). **b** Chromosome 18 regional map for reaction to isolates: OH1, OH2, OH0217, OH-Dayton, OH-MIA and OH-Windfall. Isolates designated with an “*” indicate that disease screening results from two replications were combined into a single dataset

Primers were designed for *Glyma.13G187900* at position 9025 and *Glyma.13G188300* at position 4376 (Supplementary Table S1) and mapped in three populations derived from crosses with PI 407985, PI 408097, and PI 408132 (Table 1). Both genes are classified as NLR and putative *Rps* genes for *Ph. sojae*. Hodge et al. (2025) observed that these two genes were separate from the *Rps3/Rps8* gene complex on chromosome 13 and could be novel resistance genes for the *Ph. sojae* reaction. The resultant genetic map locations of these markers (Figs. 2b, 3b and 5a) were well-supported by physical positions of the corresponding NLR genes (*Glyma.13G187900* and *Glyma.13G188300*) in the reference genome (Wm82.a4). More importantly, the chromosomal locations of these two newly developed markers coincided with the maximum LOD *Rps* response in this region on chromosome 13 (Figs. 2b, 3b and 5a).

#### Chromosome 18

Four RenSeq SNPs from NLR genes (Table 1) were added to the resistance region previously mapped to chromosome 18 in populations derived from crosses with PI 399079, PI 407985, and PI 408097. KASP genotyping primers were designed in the following four NLR genes on chromosome 18: *Glyma.18G280300* at position 0296, *Glyma.18G280366* at position 5883, *Glyma.18G281600* at position 7595, *and Glyma.18G281700* at position 6714 (Supplementary Table S1). One additional marker was developed based on unassigned chromosome 18 scaffold *Glyma.U008800* at position 608. This marker designated SF273_U8800_608, is the marker at the maximum LOD for reaction to multiple *Ph. sojae* isolates in RIL populations, PI 399079, PI 407985 and PI 408097 (Figs. 1c, 3c and 5b). The identified genetic map positions and marker order were in good agreement with the physical locations of the corresponding genes (Wm82.a4) within the respective chromosomal regions in these PIs.

### QDRL towards *Pythium* spp.

In addition to refining mapped positions for *Rps* loci, we added markers (SSRs, SNPs, and RenSeq-derived SNPs) to saturate QDRL towards *Pythium* in two populations, PI 408029 × Williams and PI 408097 × Williams, on chromosomes 6, 8 and 14 (Clevinger et al. 2021). QDRL were detected in both populations for at least two species of *Pythium* on each of these three chromosomes, so they could be useful tools in breeding programs.

**Fig. 6:**
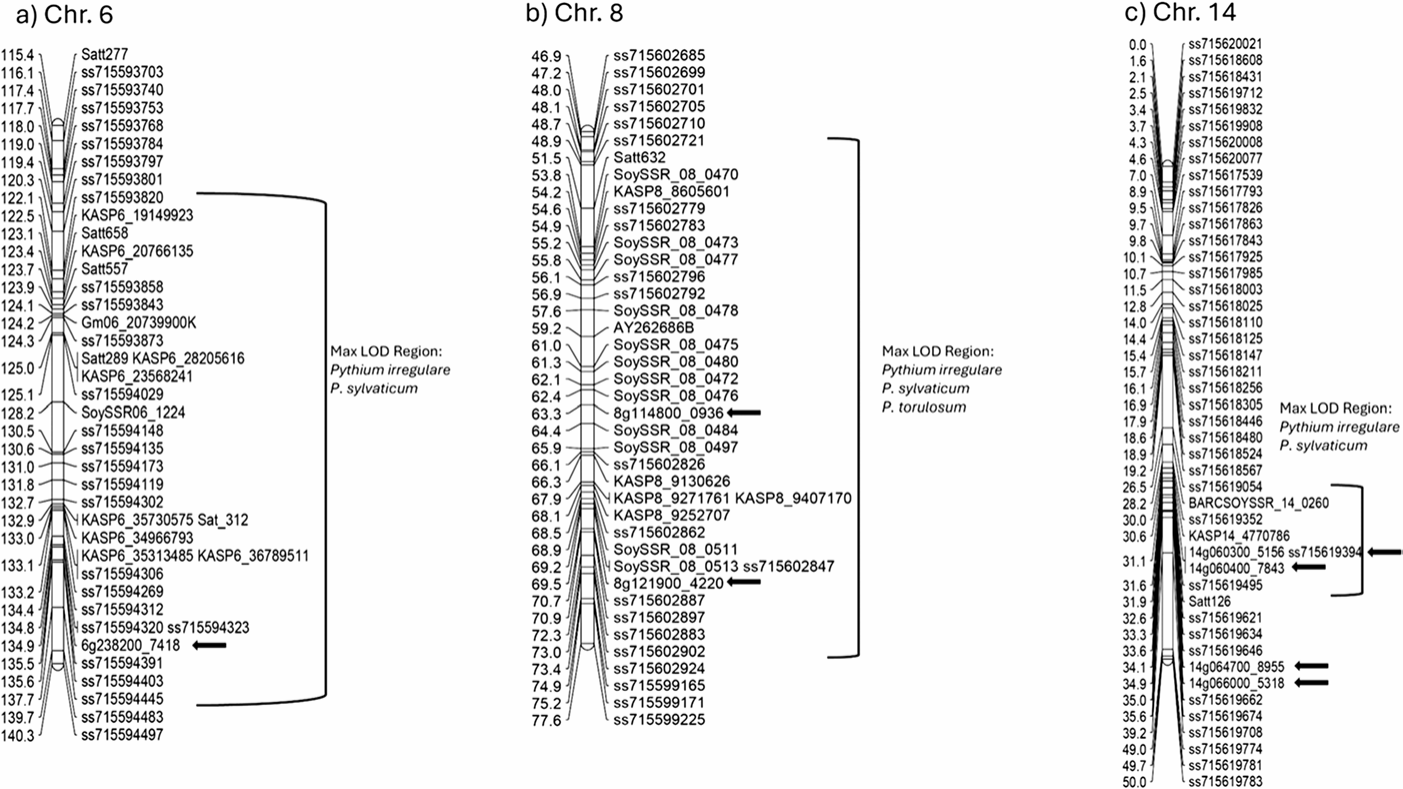
Quantitative disease resistance loci mapping in PI 408029 × Williams and PI 408097 × Williams populations towards *Pythium* species. The markers within the maximum logarithm of odds (LOD) region for reaction to specific isolates are denoted by brackets on the right side of the chromosomal map. A black arrow denotes the RenSeq-derived SNP mapped to that QDRL region. **a** Chromosome 6 regional map for PI 408029 × Williams towards resistance to *Py. irregulare* and *Py. sylvaticum*. **b** Chromosome 8 regional map for PI 408097 × Williams towards resistance to *Py. irregulare*, *Py. sylvaticum* and *Py. torulosum*. **c** Chromosome 14 regional map for PI 408029 × Williams towards resistance to *Py. irregulare* and *Py. sylvaticum*

In this study, the PI 408029 × Williams population on chromosome 6, six SSRs and twelve SNPs including one RenSeq-derived SNP (Fig. 6a, Supplementary Table S1) were added to the previous map (Clevinger et al. 2021) in this study. This large effect QDRL region is associated with resistance for the two disease reaction traits, SRS and ROTS, towards *Py. sylvaticum* and *Py. irregulare.* The RenSeq-derived SNP, 6G238200_7418, mapped within the QDRL region (Fig. 6a). *Glyma.06G238200* is a protein dephosphorylation gene located within 38,304,244–38, 308,470 bp (Wm82.a4). In the chromosome 6 QDRL region twelve gene models that were *R* gene-related sequences were identified (Supplementary Table 2 A). These are large effect but broad QDRL towards these *Pythium* spp. requiring a higher density of molecular markers. Due to the QDRL size and its impact on two necrotrophic pathogens, further studies focused on expression of genes in this region are now warranted.

In the PI 408097 x Williams population, on chromosome 8, thirteen SSRs and ten SNPs including two RenSeq-derived SNPs (Fig. 6b, Supplementary Table S1) were added to the map (Clevinger et al. 2021) in this study. These molecular markers were successfully incorporated (Fig. 6b) into the map of the PI 408097 population for the QDRL region on chromosome 8 towards *Py. sylvaticum*, *Py. irregulare* and *Py. torulosum* for disease traits, SRS and/or ROTS (Clevinger et al. 2021). This QDRL for these three *Pythium* species is important since it is also a major effect QDRL for *Py. torulosum* explaining up to 59.8% of the phenotypic variation towards resistance to this species, and those for *Py. sylvaticum* and *Py. irregulare* explained up to 22.4% and 24.9%, respectively. The two RenSeq-derived SNPs, 8G114800_0936 and 8G121900_4220, derived from *Glyma.08G114800* and *Glyma.08G121900*, respectively, were both within the QDRL region for these three *Pythium* species (Fig. 6b). The functions of *Glyma.08G114800* and *Glyma.08G121900* are annotated as a peptidyl-tRNA hydrolase and ribosome maturation factor, respectively. The physical distance between these two genes is 591,596 bp (Wm82.a4). In the QDRL region on chromosome 8, 29 disease resistance-related sequences were identified (Supplementary Table S2B). This QDRL on chromosome 8 may be useful in breeding programs since it explains a large amount of variance to multiple pathogens (Clevinger et al. 2021, 2024).

In the PI 408029 x Williams population, on chromosome 14, two SSRs and five SNPs including four RenSeq-derived SNPs (Fig. 6c) were added to the QDRL region (Clevinger et al. 2021) for disease reaction traits, SRS and/or ROTS, towards *Py. sylvaticum* and *Py. irregulare*. Two of the RenSeq SNPs, 14G060300_5156 and 14G060400_7843, associated with the leucine-rich repeat receptor-like protein kinase family genes *Glyma.14G060300* and *Glyma.14G060400*, respectively (Supplementary Table 2 C), were located within the QDRL region (Fig. 6c) for these two traits which span 25,527 bp. The other two RenSeq SNPs, 14G064700_8955 and 14G066000_5318, were located right outside of the QDRL region (Fig. 6c). We identified 31 gene models on chromosome 14 that were designated as disease resistance-related sequences (Supplementary Table S2C).

## Conclusion

The co-localization/correspondence of the developed KASP markers derived from the NLR genes from the RenSeq study mapped within the *Rps* loci and the QDRL regions of interest for *Ph. sojae* and *Pythium* species, respectively, highlights the usefulness of RenSeq developed markers for marker-assisted selection. Marker-assisted selection has improved and allowed for the acceleration of the breeding process for such traits as disease resistance by utilizing molecular markers, allowing for the screening of large populations quickly without the need for extensive phenotypic evaluation. These RenSeq derived markers provided further evidence for these novel *Rps* genes located in complex loci for *Ph. sojae*. Additionally, the saturation of the QDRL regions on chromosomes 6, 8 and 14 with KASP and other DNA markers for multiple *Pythium* species will be a useful tool in breeding resistance to these species. The markers identified in this study could be used to accelerate the breeding of resistance to these diseases.

## Supplementary Information

Below is the link to the electronic supplementary material.

Supplementary Material 1

## Data Availability

The datasets generated during and/or analyzed during the current study are available from the corresponding author on reasonable request.NBS-LRR PacBio sequencing from a Glycine max diversity panel (Hodge and Dorrance 2024)[https://doi.org/10.5061/dryad.v15dv424q](https:/doi.org/10.5061/dryad.v15dv424q).
